# Low-temperature plasma radiofrequency ablation combined with multimodal bronchoscopic interventions for tracheobronchial amyloidosis-induced airway stenosis: a case report and literature review

**DOI:** 10.3389/fmed.2026.1972108

**Published:** 2026-10-07

**Authors:** Rongrong Zhou, Huaxu Yin, Xing Chen, Wenjie Wu, Xin Xiong, Jie Zhang, Ting Wang, Bing Xue

**Affiliations:** Department of Respiratory and Critical Care Medicine, Chuiyangliu Hospital Affiliated to Tsinghua University, Beijing, China

**Keywords:** airway stenosis, individualized treatment, low-temperature plasma radiofrequency ablation, multimodal bronchoscopic intervention, tracheobronchial amyloidosis

## Abstract

Tracheobronchial amyloidosis (TBA) is a rare airway disorder characterized by abnormal deposition of amyloid protein in the submucosa and muscular layer of the tracheobronchial tree. Clinical manifestations are nonspecific, and the disease is frequently misdiagnosed as chronic obstructive pulmonary disease, bronchial asthma, or airway malignancy. No standardized diagnostic and treatment guidelines exist, and bronchoscopic interventions have become the preferred approach for relieving airway obstruction. We report a 56-year-old man with TBA presenting with a 12-year history of recurrent cough, sputum production, and dyspnea. Chest computed tomography (CT) revealed diffuse bronchial wall thickening with calcification and irregular luminal narrowing, with obstructive pneumonia and atelectasis in the right middle and lower lobes. Bronchoscopy demonstrated diffuse mucosal thickening with nodular hyperplasia and marked luminal stenosis. Congo red staining was positive with apple-green birefringence under polarized light, confirming localized TBA. The patient underwent three stages of multimodal bronchoscopic intervention over 12 months—comprising balloon dilatation, forceps debridement, diode laser ablation, low-temperature plasma radiofrequency ablation (LTP-RFA), and cryotherapy—to treat the primary, recurrent, and residual airway stenosis. Following serial treatments, the patient's symptoms improved significantly, with endoscopic and radiographic improvement. This case represents one of the few reports of LTP-RFA application in TBA. We review the recent literature on TBA treatment modalities and outcomes, providing a reference for individualized TBA management.

## Introduction

Tracheobronchial amyloidosis (TBA) is characterized by the extracellular deposition of misfolded amyloid fibrils in the submucosa and muscular layer of the tracheobronchial tree, ultimately leading to airway stenosis, occlusion, and progressive pulmonary impairment (1, 2). Respiratory amyloidosis comprises three main types: TBA, nodular pulmonary amyloidosis (NPA), and diffuse pulmonary amyloidosis (DPA), with TBA being the most common (3–5). The disease has an extremely low incidence, with only several hundred cases reported worldwide (2, 6). Its onset is insidious and clinical manifestations lack specificity, frequently resulting in misdiagnosis as chronic obstructive pulmonary disease, bronchial asthma, airway malignancy, or pulmonary tuberculosis (6, 7).

Currently, no unified diagnostic and treatment guidelines exist for TBA. The therapeutic goals focus on alleviating clinical symptoms, relieving airway obstruction, and delaying disease progression (5). Treatment options include bronchoscopic interventions, external beam radiotherapy (EBRT), watchful waiting, systemic therapy, and surgery, with management strategies primarily based on case reports and expert opinion (8). According to a systematic review by Smesseim et al. (8) published in 2024 (including 77 studies and 300 patients), bronchoscopic interventions accounted for 30.3% of treatments (Nd:YAG laser 26.3%, mechanical debulking 8.0%, argon plasma coagulation 3.7%, cryotherapy 1.3%, stent placement 1.7%, balloon dilatation 1.7%), EBRT 22.0%, watchful waiting 21.7%, systemic therapy 16.3%, and surgery 4.0%. Given its minimally invasive nature and definitive efficacy, bronchoscopic intervention has become the preferred approach for relieving airway obstruction in TBA (1, 9). However, the overall recurrence rate remains high, and most patients require repeated interventions to maintain therapeutic efficacy (1, 8).

Low-temperature plasma radiofrequency ablation (LTP-RFA) is a novel minimally invasive thermal ablation technique that operates at 40–70 °C. It utilizes bipolar radiofrequency energy to excite a saline medium into a plasma field, specifically cleaving tissue peptide bonds to achieve low-temperature ablation with concurrent hemostasis, while minimizing thermal injury to surrounding normal tissues (10, 11). This technology has been applied in benign central airway stenosis (12, 13) and laryngotracheal stenosis (14), but reports of its use in TBA are exceedingly rare, limited to a single case of subglottic amyloid stenosis by Kosnik et al. (15) in 2023. We report a case of TBA-induced airway stenosis successfully treated with LTP-RFA combined with multimodal bronchoscopic interventions and provide a narrative literature review of TBA characteristics, diagnostic framework, and current bronchoscopic treatment modalities to inform individualized management.

## Case presentation

### Patient information

A 56-year-old man was admitted on November 12, 2024, with a 12-year history of recurrent cough, sputum production, and dyspnea that had worsened for over one month. Twelve years prior, he developed cough with yellow-green viscid sputum, positional sputum production, and exertional dyspnea, worsening in autumn and winter, partially relieved by antibiotics, expectorants, and irregular tiotropium inhalation. One month prior to admission, symptoms worsened with occasional blood-tinged sputum, and antibiotic treatment at an outside hospital was ineffective. His past medical history included type 2 diabetes mellitus for over 10 years on regular antidiabetic therapy; a 30-year smoking history (60 cigarettes/day), having quit 10 years prior; alcohol consumption for 30 years (approximately 500 mL liquor/day), having abstained for 10 years; and a 10-year history of cast iron dust exposure.

### Clinical findings

Physical examination: temperature 36.0 °C, heart rate 76 beats/min, respiratory rate 18 breaths/min, blood pressure 106/77 mmHg; conscious and alert, no cyanosis; decreased breath sounds in the right lung with moist crackles; regular heart rhythm without pathological murmurs; no lower extremity edema or digital clubbing. Laboratory tests: white blood cell count 4.5 × 10^9^/L, platelet count 120 × 10^9^/L, C-reactive protein 107.04 mg/L; arterial blood gas analysis: pH 7.37, PaO_2_ 93.3 mmHg, PaCO_2_ 33.2 mmHg, SaO_2_ 96.8%; erythrocyte sedimentation rate 17 mm/h, procalcitonin 0.24 ng/mL; serum protein electrophoresis and monoclonal immunoglobulin testing were unremarkable; autoantibodies negative; purified protein derivative (PPD) test and interferon-gamma release assay negative; sputum microbiology and acid-fast bacilli staining negative; tumor markers negative. Electrocardiogram and echocardiography were unremarkable.

### Imaging and bronchoscopic findings

Cervicothoracic CT (November 12, 2024; Figures 1a,b) demonstrated diffuse bronchial wall thickening with calcification and irregular luminal narrowing, with nodular protrusions in some segments. The right middle and lower lobes were most severely affected, with obstructive pneumonia, atelectasis, and bronchiectasis with infectious infiltrates in the right lower lobe.

**Figure 1:**
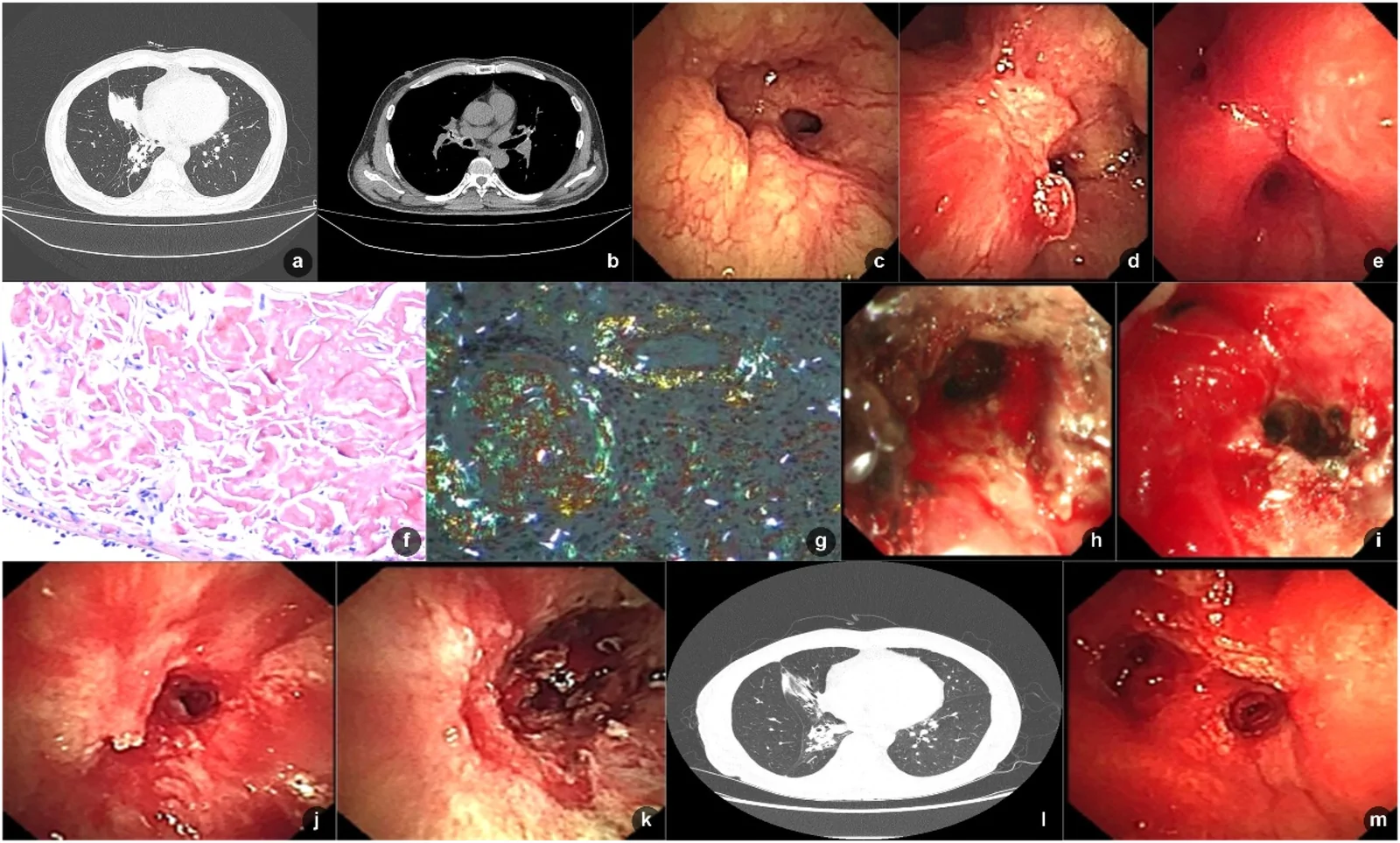
**(a,b)** cervicothoracic CT (November 12, 2024): diffuse bronchial wall thickening with calcification and irregular luminal narrowing, with nodular protrusions; the right middle and lower lobes are most affected, with obstructive pneumonia, atelectasis, and right lower lobe bronchiectasis with infectious infiltrates. **(c–e)** Bronchoscopy (November 13, 2024): Diffuse mucosal thickening and nodular hyperplasia in the right upper lobe **(c)**, right intermediate bronchus **(d)**, and lower lobe **(e)**, with marked luminal stenosis preventing bronchoscope passage. **(f)** Positive Congo red staining. **(g)** Apple-green birefringence under polarized light. **(h,i)** Balloon dilatation of the right middle lobe followed by diode laser ablation of the right middle and lower lobes (November 18, 2024). **(j,k)** After LTP-RFA treatment of the right middle **(j)** and lower **(k)** lobe bronchi (November 25, 2024). **(l)** Follow-up chest CT showing improvement of right middle lobe obstructive pneumonia and partial lung re-expansion, with partial improvement of right lower lobe bronchiectasis and infection (November 26, 2024). **(m)** Right intermediate bronchus after forceps debridement (December 12, 2024).

Flexible therapeutic bronchoscopy (November 13, 2024) revealed smooth mucosa and patent lumen in the left main bronchus, with thickened carina between the left upper lobe and lingula and narrowed orifices. The left lower lobe remained patent. In the right lung, the main bronchial mucosa was rough, with diffuse thickening, nodular hyperplasia, and marked luminal stenosis in the upper, middle, and lower lobe bronchi, preventing bronchoscope passage (Figures 1c–e). Mucosal biopsy of the right lower lobe basal segment was performed. Histopathology showed chronic bronchial mucosal inflammation with sheet-like homogeneous eosinophilic deposits in the stroma; Congo red staining was positive (Figure 1f), with apple-green birefringence under polarized light (Figure 1g). Combined with clinical, radiographic, and pathological findings, and after excluding tracheobronchial tuberculosis, airway malignancy, sarcoidosis, and tracheobronchopathia osteochondroplastica (TPO), a definitive diagnosis of TBA was established. TPO was considered unlikely: although TPO can also manifest as calcified submucosal nodules with diffuse airway wall thickening on CT, it characteristically spares the posterior membranous wall of the trachea, whereas the airway wall thickening in our patient was circumferential and involved the posterior membranous wall; moreover, histopathology in TPO reveals submucosal osteocartilaginous nodules rather than congophilic amyloid deposits (16, 17). Serum protein electrophoresis and monoclonal immunoglobulin testing were unremarkable, and systemic evaluation revealed no evidence of other organ involvement, confirming localized TBA (1, 4, 9).

### Therapeutic intervention

The patient underwent multistage, multimodal bronchoscopic intervention under general anesthesia (propofol, sufentanil, rocuronium) via a rigid bronchoscope with jet ventilation, through which a flexible therapeutic bronchoscope was advanced.

**Key equipment and parameters were as follows**: flexible therapeutic bronchoscope (Aohua VBC-T300; outer diameter 4.9 mm, working channel 3.0 mm); diode laser system (LaserPro D980; 980 nm wavelength, maximum output 25 W), used in non-contact mode at 10 W with 0.5–2.0 s pulses; cryotherapy system (ERBE ERBOKRYO CA; N_2_O-based, flexible cryoprobe 2.4 mm outer diameter; effective tissue temperature −70 °C, 3-min freeze cycles with auto-thaw); LTP-RFA system (BONSS ARS600 with AC403 electrode; approximately 100 kHz, 40–70 °C, ablation 7 W, coagulation 3 W, saline irrigation 80–100 drops/min); and balloon dilatation catheter (Boston Scientific CRE; three-stage 10/11/12 mm, maximum inflation 2.5 atm for 30 s × 3).

The treatment strategy was individualized according to the lesion characteristics at each stage. Because the amyloid deposits were diffuse and multisegmental and the amyloid tissue was fragile and prone to bleeding, a single technique was unlikely to achieve durable airway recanalization. A sequential multimodal strategy was therefore adopted: balloon dilatation and forceps debridement for rapid restoration of luminal patency, diode laser ablation for rapid debulking of nodular and circumferential lesions, LTP-RFA for precise low-temperature ablation of diffuse mucosal thickening with integrated hemostasis, and cryotherapy for consolidation of the treated segments and delayed devitalization of residual deposits.

#### Stage 1 (November 18-25, 2024)

On November 18 (Figures 1h,i), balloon dilatation was performed at the right middle lobe bronchus orifice (2.5 atm, 30 s × 3), resulting in improved luminal patency. Forceps debridement of hyperplastic tissue at the right middle lobe orifice was then performed, followed by diode laser ablation of the thickened mucosa in the right upper lobe, middle lobe, and lower lobe basal and posterior segments. After treatment, distal bronchi of the right middle and lower lobes became visible, and copious purulent, blood-tinged sputum was aspirated. On November 21, necrotic tissue was debrided from the right intermediate bronchus and right lower lobe bronchus using biopsy forceps; significant intraoperative bleeding was controlled with ice-cold saline, and purulent secretions were aspirated from distal bronchi. On November 25, LTP-RFA was applied to the thickened mucosa of the right middle lobe (Figure 1j) and lower lobe (Figure 1k) bronchi; the ablation electrode was moved slowly along the mucosal surface for point-by-point ablation of thickened mucosa and nodular hyperplasia, with minimal intraoperative bleeding and further improvement in airway stenosis (12, 15). Follow-up chest CT on November 26 showed improvement of right middle lobe obstructive pneumonia with partial lung re-expansion, and partial improvement of right lower lobe bronchiectasis and infection (Figure 1l). On December 12, follow-up bronchoscopy was performed with debridement of necrotic tissue from the right intermediate bronchus and aspiration of copious purulent secretions from distal bronchi (Figure 1m).

#### Stage 2 (March 26-April 7, 2025)

Follow-up chest CT on March 24, 2025 showed further re-expansion of the right middle lobe compared with November 26, 2024, but progression of right lower lobe infection with atelectasis (Figure 2a). Bronchoscopy on March 26 revealed mucosal thickening and luminal stenosis in the right upper lobe (Figure 2b), with thickened, edematous walls and marked stenosis at the orifices of the right middle and lower lobe bronchi (Figure 2c). Given that the recurrent stenosis was again dominated by mucosal thickening and edema, laser ablation was selected for rapid debulking, and cryorecanalization was added to consolidate the recanalized segments and delay re-proliferation. On March 31, diode laser ablation was performed on the thickened mucosa of the right upper lobe posterior segment (Figure 2d) and anterior segment, right middle lobe, and right lower lobe basal segments (Figure 2e), with forceps-assisted debridement, resulting in significant improvement of luminal stenosis. On April 3, necrotic tissue was debrided from the right upper lobe and right intermediate bronchus, with aspiration of copious gelatinous secretions. On April 7, cryorecanalization was performed in the right upper lobe apical segment, middle lobe, and lower lobe using a cryoprobe for repeated freeze-thaw cycles, with visible improvement in bronchial stenosis (Figures 2f–h).

**Figure 2:**
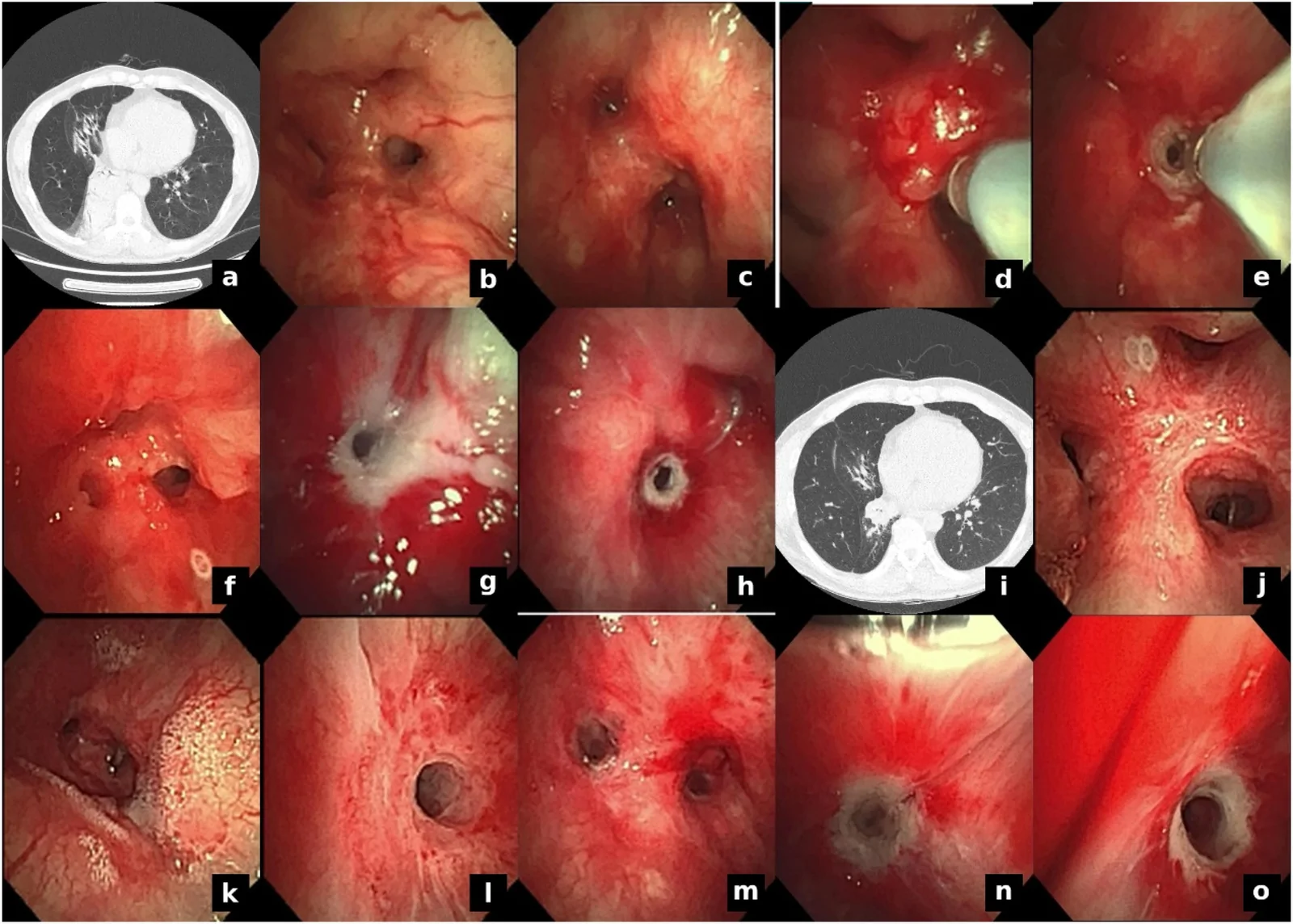
**(a)** follow-up chest CT (March 24, 2025): further re-expansion of the right middle lobe compared with November 26, 2024, but progression of right lower lobe infection with atelectasis. **(b,c)** Bronchoscopy (March 26, 2025): Mucosal thickening and luminal stenosis in the right upper lobe **(b)**, with thickened, edematous walls and marked stenosis at the right middle and lower lobe bronchial orifices **(c). (d,e)** Diode laser ablation of the right upper lobe posterior segment **(d)** and right lower lobe basal segment **(e)** mucosal thickening (March 31, 2025). **(f–h)** Cryorecanalization of the right upper lobe apical segment, middle lobe, and lower lobe, with improved bronchial stenosis (April 7, 2025). **(i)** Follow-up chest CT (November 3, 2025) compared with March 2025: Further re-expansion of the right middle lobe and re-expansion of the right lower lobe. **(j–l)** Bronchoscopy (November 5, 2025): Improved luminal stenosis in the right upper lobe segmental bronchi **(j)**, but mucosal infiltration and deformation with pinhole-like stenosis in the right intermediate bronchus **(k)** and lower lobe basal segments **(l)**. **(m–o)** LTP-RFA treatment of the right middle lobe **(n)** and lower lobe basal segments **(o)**, with improved luminal patency, combined with local cryotherapy (November 10, 2025).

#### Stage 3 (November 5-10, 2025)

Bronchoscopy on November 5 showed improved luminal stenosis in the right upper lobe segmental bronchi compared with before treatment (Figure 2j), but the right intermediate bronchus (Figure 2k) and lower lobe basal segments (Figure 2l) showed mucosal infiltration and deformation with pinhole-like stenosis. Because the residual stenosis was caused predominantly by diffuse mucosal infiltration rather than bulky intraluminal disease, LTP-RFA combined with cryotherapy—rather than repeated laser debulking—was selected to ablate the thickened mucosa precisely at low temperature with a low risk of bleeding. On November 10, LTP-RFA was applied to the orifices of the right middle lobe (Figure 2n), lower lobe basal segments (Figure 2o), and upper lobe anterior segment bronchi, with improved luminal patency, combined with local repeated cryotherapy (freeze-thaw) (Figures 2m–o).

During each hospitalization, the patient received supportive treatment including antibiotics, expectorants, and bronchodilators.

### Follow-up and outcomes

Follow-up chest CT on November 3, 2025 (Figure 2i) reported: compared with the previous examination (March 24, 2025), further re-expansion of the right middle lobe and re-expansion of the right lower lobe, with resolution of the right lower lobe atelectasis and improvement of the infectious infiltrates; the diffuse tracheobronchial wall thickening with calcification persisted, consistent with the known diagnosis of TBA. The final bronchoscopic assessment (November 5, 2025) demonstrated improved luminal stenosis in all segmental bronchi compared with before treatment, with visible distal bronchi. After serial treatments, the patient's cough and sputum production improved significantly, with reduced sputum volume and markedly decreased dyspnea. At telephone follow-up in April 2026, the patient reported occasional cough and sputum production with no significant dyspnea. Of note, pulmonary function testing and standardized bronchoscopic stenosis grading were not performed during follow-up; the efficacy evaluation therefore relied mainly on symptomatic improvement and qualitative imaging and endoscopic findings, and the conclusions regarding treatment efficacy should be interpreted with this limitation in mind. The diagnostic and treatment timeline is summarized in Table 1.

**Table 1:** Diagnostic and treatment timeline.

Date	Event	Examination/Treatment	Outcome
2024-11-12	Admission	Cervicothoracic CT, bronchoscopy+biopsy	TBA confirmed
2024-11-18	First intervention	Balloon dilatation+forceps debridement+laser ablation	Luminal stenosis improved
2024-11-21	Post-operative review	Necrotic tissue debridement	Airway patency improved
2024-11-25	LTP-RFA	Low-temperature plasma radiofrequency ablation	Airway stenosis further improved
2024-11-26	Follow-up	Chest CT	Obstructive pneumonia reduced; atelectasis improved
2024-12-12	Post-operative review	Necrotic tissue debridement	Airway patency improved
2025-03-24	Recurrence follow-up	Chest CT	Right lower lobe infection with atelectasis progressed
2025-03-26	Recurrence assessment	Bronchoscopy	Luminal re-stenosis
2025-03-31 to 04-07	Re-intervention	Laser ablation+forceps debridement+cryorecanalization	Luminal stenosis significantly improved
2025-11-03	Follow-up	Chest CT	Partial improvement; right lower lobe re-expansion
2025-11-10	LTP-RFA+cryotherapy	LTP-RFA+cryotherapy (freeze-thaw)	Luminal stenosis improved
2026-04	Telephone follow-up	Symptom assessment	Occasional cough; no significant dyspnea

## Discussion

### TBA disease characteristics and treatment challenges

TBA is the most common form of respiratory amyloidosis. Its core pathological feature is amyloid deposition in the submucosa and muscular layer of the tracheobronchial tree, resulting in airway wall thickening and luminal narrowing (3, 4). Most TBA cases present as localized AL-type amyloidosis (1, 4, 9), confined to the airways with low risk of systemic involvement. Clinical symptoms correlate with the location, extent, and severity of deposition, primarily manifesting as cough, sputum production, dyspnea, and hemoptysis (7, 11). Airway stenosis leads to secretion retention, frequently complicated by infection and atelectasis. TBA should also be differentiated from TPO, which similarly produces calcified submucosal nodules and diffuse airway wall thickening but characteristically spares the posterior membranous wall of the trachea, whereas amyloid deposition in TBA involves the entire airway circumference, including the posterior membranous wall (16, 17). The pathogenesis remains incompletely understood, though smoking and dust exposure may contribute through chronic airway mucosal injury, immune dysregulation, and protein misfolding (2, 18, 19). Our patient had a 30-year heavy smoking history and 10-year cast iron dust exposure, suggesting that occupational exposure and adverse lifestyle habits may be important contributing factors.

The treatment challenges of TBA include: (1) disease rarity, with lack of large-sample randomized controlled trials and unified guidelines (8); (2) diffuse, multisegmental involvement that is difficult to completely eradicate; (3) high overall recurrence rate, with most patients requiring repeated interventions (1, 8, 20); and (4) fragile amyloid tissue prone to bleeding, compromising the operative field and procedural safety (20). O’Regan et al. (6) reported the Boston University experience with 10 TBA patients, where repeated rigid bronchoscopic debulking and laser therapy failed to prevent progressive airway stenosis in advanced cases, with 30% mortality within 7–12 years of diagnosis, underscoring the formidable challenges of TBA management.

### Bronchoscopic intervention modalities for TBA

Bronchoscopic intervention has become the preferred treatment for TBA. According to the systematic review by Smesseim et al. (8), therapeutic bronchoscopy accounted for 30.3% of all TBA treatments. Lu et al. (7) retrospectively analyzed 109 Chinese TBA patients, of whom 53 underwent bronchoscopic interventions, with improvement in 51 cases, demonstrating good short-term efficacy. However, each interventional technique has specific indications and limitations (Table 2). Clinical practice often requires a multi-technique combination strategy based on lesion extent, stenosis severity, and bleeding risk (8, 21–23).

**Table 2:** Comparison of bronchoscopic intervention modalities for TBA.

Modality	Indications	Advantages	Limitations
Nd:YAG laser ablation	Tracheal/main bronchial obstruction; lesions <4 cm	Most commonly used; immediate airway recanalization; deep lesion penetration; effective hemostasis (15–40 W)	Significant thermal injury (>100 °C); airway fire risk (FiO_2_ < 40% required); expensive; deep penetration may damage cartilage; high recurrence
Mechanical debulking (rigid bronchoscope)	Large intraluminal nodules/masses; rapid obstruction relief	Rapid bulk removal; no thermal injury; combinable with other techniques	Requires general anesthesia with rigid bronchoscope; high bleeding risk (fragile amyloid tissue); limited efficacy for diffuse lesions
Argon plasma coagulation (APC)	Superficial lesion ablation; hemostasis; residual tissue clearance	Effective hemostasis; non-contact; suitable for diffuse superficial lesions (20–40 W)	Shallow penetration (2–3 mm); ineffective for large masses alone; FiO_2_ < 40% required; potential granulation; gas embolism risk
Cryotherapy	Residual lesions; diffuse mucosal disease; cryobiopsy	Preserves tissue for pathology; no thermal injury; protects cartilage; repeatable; low cost	Limited immediate effect; slow onset (freeze-thaw cycles); unsuitable for acute obstruction; requires multiple sessions
Balloon dilatation	Luminal stenosis (non-mass predominant); post-stent dilatation	Immediate airway expansion; simple; repeatable	Re-stenosis when used alone (1/3–1/2 require additional treatment); less effective for thickened/calcified walls; airway tear risk
Stent placement	Recurrent stenosis; bronchomalacia; post-dilatation re-stenosis	Maintains airway patency; improves quality of life; combinable	Granulation, migration, secretion retention; requires replacement/removal; high recurrence in TBA
High-frequency electrocautery	Local lesion cutting; fibrotic band incision	Can incise fibrotic bands; widely available; low cost	Thermal injury risk (400-600 °C); may exacerbate scarring; carbonization; cartilage damage risk
LTP-RFA	Diffuse mucosal thickening; nodular hyperplasia; recurrent refractory stenosis	Low working temperature (40–70 °C); minimal thermal injury; integrated ablation-hemostasis; very low airway fire risk	Limited TBA evidence (case reports only); relatively large probe; requires combination for severe stenosis; saline medium required

Utilization rates are cited from Smesseim et al. (8) (300 patients). Nd:YAG laser and APC parameters are referenced from Sachdeva et al. (24) and Reichle et al. (25); cryotherapy parameters from ERBE ERBOKRYO CA specifications and Jeong et al. (26); balloon parameters from Boston Scientific CRE product specifications and Sachdeva et al. (24); LTP-RFA parameters from Li et al. (12) and BONSS ARS600 product specifications.

### LTP-RFA: advantages, limitations, and combination strategy

LTP-RFA operates at 40–70 °C, far below conventional thermal ablation techniques (laser >100 °C, APC approximately 100–150 °C, high-frequency electrocautery 400–600 °C) (12, 14). It achieves ablation through plasma-field-mediated chemical cleavage of tissue peptide bonds rather than high-temperature cauterization, resulting in minimal thermal injury to adjacent normal tissues (10, 12). A comparative study by Hao et al. (14)demonstrated that, compared with CO_2_ laser, LTP-RFA offered significant advantages in operative time, intraoperative blood loss, postoperative pain scores, and certain complication rates.

The advantages of LTP-RFA for TBA include: (1) low-temperature ablation significantly reduces thermal injury to the airway mucosa and the risk of post-procedural scar formation, and is particularly suitable for thermosensitive tracheobronchial tissues (12); (2) combined ablation and hemostasis functions address the fragile, hemorrhage-prone nature of amyloid tissue, ensuring procedural precision and safety with minimal intraoperative bleeding (12, 15); and (3) precise operation with adaptable probe tips allows targeted ablation of diffusely thickened mucosa and nodular hyperplasia (15).

However, LTP-RFA has limitations: (1) current evidence for TBA is limited to case reports (15), lacking large-sample validation of long-term efficacy and optimal parameters; (2) for large deposits or severe luminal stenosis, it cannot rapidly establish airway patency alone and requires initial debulking with laser or rigid bronchoscope (12); (3) the relatively large probe tip limits maneuverability in distal small airways; and (4) like all endoscopic techniques, it cannot eradicate the underlying submucosal amyloid deposition, with persistent recurrence risk.

The combination strategy offers multi-technique complementarity: in our case, LTP-RFA was combined with laser ablation, balloon dilatation, and cryotherapy. Li et al. (12) used the BONSS ARS600 system combined with balloon dilatation and stent placement in 32 patients with benign central airway stenosis, with all patients achieving stenosis relief without significant complications. Virk et al. (13) demonstrated the feasibility of plasma ablation as an alternative tool for managing postintubation laryngotracheal stenosis. The combination strategy leverages each technique's strengths to improve lesion clearance efficiency and is particularly suitable for diffuse, multisegmental TBA.

### Literature review of TBA cases

We reviewed TBA cases with bronchoscopic intervention reported over the past decade (2014–2025). Sommer et al. (22) reported two TBA patients treated with rigid bronchoscopic Nd:YAG laser debulking and EBRT (20 Gy/10 fractions); at 2-year and 9-month follow-up, respectively, one patient remained asymptomatic and the other had intermittent dyspnea that improved compared with baseline. Lu et al. (7) reported 109 Chinese TBA patients (53 undergoing bronchoscopy), with 51 of 53 showing improvement; among patients followed for more than 3 months, 2 improved, 47 remained stable, and 9 worsened. Zhang et al. (20) reported 12 TBA patients treated with various bronchoscopic modalities, with dyspnea scores improving from 3.3 to 1.2 (*P* < 0.05), though 2 patients experienced massive hemorrhage. Arulanantham et al. (23) reported a localized TBA case managed with cryodebridement, balloon dilatation, metallic stent, EBRT, and silicone stent, with no evidence of active disease at 6-month follow-up. Yan et al. (9) reported a TBA case treated with APC and Nd:YAG laser followed by EBRT (ineffective), ultimately requiring CyBorD chemotherapy (cyclophosphamide, bortezomib, dexamethasone); ECOG performance status improved from 4 to 0 and FEV_1_ from 18.9% to 63.8% predicted, though bronchoscopic obstruction remained unchanged. Kosnik et al. (15) reported LTP-RFA treatment of subglottic amyloid stenosis with improved airway patency and no serious complications. Shterev et al. (2) reported a case managed by complete surgical resection of a subglottic mass, with 3-year disease-free survival followed by multiple recurrences.

The systematic review by Smesseim et al. (8) highlighted the highly heterogeneous treatment landscape for TBA, with no randomized controlled trials and treatment decisions based on expert opinion, noting that local resection often provides only temporary symptomatic improvement with multiple recurrences. These reports collectively emphasize the need for individualized, multimodal treatment approaches in TBA, and underscore that bronchoscopic interventions, while effective for short-term symptom relief, rarely achieve durable disease control without repeated sessions or multimodal combination strategies.

### Assessment metrics and clinical decision-making

No standardized efficacy assessment system exists for TBA treatment. Based on the literature, a multidimensional assessment framework is recommended, including: (1) symptomatic assessment using mMRC dyspnea scale, ECOG performance status, and evaluation of cough, hemoptysis, and wheezing relief (9, 20); (2) pulmonary function testing (FEV_1_, FVC, FEV_1_/FVC, flow-volume loops), with O’Regan et al. (6) noting that serial pulmonary function tests are the most sensitive indicator of disease progression; (3) imaging assessment using chest HRCT for quantitative evaluation of airway narrowing, wall thickening, and secondary parenchymal changes (5, 6); (4) bronchoscopic assessment of airway patency, lesion extent, and mucosal appearance, which is the core modality for TBA diagnosis, treatment, and follow-up (2, 8); and (5) inflammatory markers including ESR and CRP. Smesseim et al. (8) noted that pre- and post-treatment symptomatic and pulmonary function data in the literature are very limited, representing a major evidence gap.

No formal clinical practice guidelines exist for TBA (8). The WABIP guidelines on airway stenting (27) provide a framework for bronchoscopic intervention in airway stenosis but primarily address malignant central airway obstruction, without fully addressing the specific characteristics of TBA as a benign, rare etiology. Based on current evidence and our experience, we recommend the following clinical decision-making principles: (1) individualized, stratified treatment based on symptom severity, airway obstruction degree, and lesion extent (8); (2) watchful waiting with CT and bronchoscopy every 6–12 months for asymptomatic or localized disease (8); (3) minimally invasive interventions (balloon dilatation, cryotherapy, forceps debridement) for mild-to-moderate obstruction; (4) multimodal combination strategy for moderate-to-severe, diffuse, or recurrent disease, with LTP-RFA as the preferred ablation modality for diffuse mucosal lesions (12, 15); (5) concurrent systemic supportive treatment (antibiotics, expectorants, bronchodilators); and (6) long-term follow-up with bronchoscopy and pulmonary function testing every 3 months post-intervention (8, 20).

### Novelty, limitations, and clinical implications

Compared with previous reports, this case demonstrates several unique aspects: (1) it is among the first to systematically apply LTP-RFA for diffuse TBA-induced airway stenosis, expanding the application scope beyond the subglottic case reported by Kosnik et al. (15); (2) it establishes a five-modality combination strategy (LTP-RFA, laser ablation, balloon dilatation, cryotherapy, forceps debridement), better suited to the diffuse, multisegmental pathology of TBA than single- or dual-technique approaches (9, 22, 23); (3) it documents the complete recurrence and re-intervention process, providing a reproducible clinical pathway for TBA recurrence management; and (4) it provides mid-term efficacy data with a follow-up exceeding 12 months.

Limitations include: (1) single-case report with limited generalizability; (2) incomplete assessment metrics, lacking systematic pre- and post-treatment pulmonary function testing (FEV_1_, FVC), standardized dyspnea scores, and quantitative bronchoscopic stenosis grading, with primarily qualitative imaging and endoscopic assessment, which weakens the objectivity and credibility of the efficacy conclusions; (3) limited follow-up duration (currently over 12 months), with long-term efficacy and recurrence rates requiring further observation; (4) the final follow-up was by telephone, as the patient declined repeat CT and bronchoscopy, lacking objective efficacy data; and (5) no control group for direct comparison with other interventional approaches. Future multicenter, prospective studies with standardized assessment metrics are needed to determine the optimal indications, parameters, and long-term outcomes of LTP-RFA in TBA.

For interventional pulmonologists, this case highlights: (1) the characteristic bronchoscopic appearance of TBA (diffuse mucosal thickening, nodular hyperplasia, luminal stenosis), warranting early inclusion in the differential diagnosis for patients with refractory respiratory symptoms and airway stenosis (1, 6, 7); (2) multi-site, deep biopsy with Congo red staining and polarized light birefringence required for definitive diagnosis (1, 27); (3) a debulk-first, ablate-second, consolidate-third strategy, with initial rapid debulking using laser or rigid bronchoscope, followed by LTP-RFA for precise ablation of diffuse mucosal thickening, and cryotherapy for consolidation; (4) the significant advantage of LTP-RFA’s concurrent hemostasis given the vascular, fragile nature of amyloid tissue (12, 20); and (5) the necessity of long-term follow-up with close recurrence monitoring and timely re-intervention (8).

## Conclusion

TBA is a rare airway disorder whose diagnosis relies on the triad of clinical symptoms, imaging, and histopathological biopsy. Treatment focuses on relieving airway obstruction, controlling symptoms, and delaying progression. This case demonstrates that LTP-RFA, as a novel minimally invasive ablation technique, offers unique advantages in low-temperature safety, precise ablation, and concurrent hemostasis, and combined with multimodal bronchoscopic intervention can effectively improve TBA-induced diffuse airway stenosis. However, clinical reports of LTP-RFA for TBA remain limited, and its long-term efficacy, optimal parameters, and recurrence rates require further validation. Multicenter collaborative studies with large-sample data are needed to establish the indications, procedural standards, and long-term outcomes of LTP-RFA, advancing individualized and standardized TBA management.

## Patient perspective

The patient reported significant improvement in cough and sputum production, with markedly reduced sputum volume and dyspnea, and improved daily living capacity after serial bronchoscopic interventions. He was generally satisfied with the minimally invasive treatment outcomes but expressed some psychological burden regarding the need for repeated treatments and disease recurrence. Written informed consent was obtained from the patient for publication of this case report.

## Data Availability

The original contributions presented in the study are included in the article/Supplementary Material, further inquiries can be directed to the corresponding authors.

## References

[1] Riehani A Soubani AO. The spectrum of pulmonary amyloidosis. Respir Med. (2023) 218:107407. 10.1016/j.rmed.2023.107407 37696313

[2] Shterev F Aleksiev V Chonov V Yavorov B Kartev S Argirov D. Tracheobronchial amyloidosis: a rare airway disorder with diagnostic and therapeutic challenges-A. *Case Report and Literature Review*. Respirol Case Rep. (2025) 13(4):e70171. 10.1002/rcr2.70171 40231310 PMC11994858

[3] Milani P Basset M Russo F Foli A Palladini G Merlini G. The lung in amyloidosis. Eur Respir Rev. (2017) 26(145):170046. 10.1183/16000617.0046-2017 28877975 PMC9488920

[4] Khoor A Colby TV. Amyloidosis of the lung. Arch Pathol Lab Med. (2017) 141(2):247–54. 10.5858/arpa.2016-0102-ra 28134587

[5] Khatalin M A Dalal A Ozgen KH Yildirim O. Tracheobronchial amyloidosis: a case report. Indian J Otolaryngol Head Neck Surg. (2023) 75(2):1024–7. 10.1007/s12070-022-03185-z 37274967 PMC10234968

[6] O'Regan A Fenlon HM Beamis JF Steele MP Skinner M Berk JL. Tracheobronchial amyloidosis. The Boston university experience from 1984 to 1999. Medicine (Baltimore). (2000) 79(2):69–79. 10.1097/00005792-200003000-00001 10771705

[7] Lu X He B Wang G He B Wang L Chen Q. Bronchoscopic diagnosis and treatment of primary tracheobronchial amyloidosis: a retrospective analysis from China. Biomed Res Int. (2017) 2017:3425812:1–7. 10.1155/2017/3425812 PMC5288517 28197412

[8] Smesseim I Cobussen P Thakrar R Daniels H. Management of tracheobronchial amyloidosis: a review of the literature. ERJ Open Res. (2024) 10(1):00540-2023. 10.1183/23120541.00540-2023 38333645 PMC10851947

[9] Yan W Li P Wu C Zhou C Liao A Yang W. Case report: management of primary tracheobronchial light chain amyloidosis in a patient with biclonal gammopathy using a systemic bortezomib-based regimen. Front Med (Lausanne). (2021) 8:728561. 10.3389/fmed.2021.728561 34722570 PMC8554224

[10] Zhang K Xu Y Yin T Ji F Xu H. A study of the safety of low temperature plasma radiofrequency ablation on rat sciatic nerve. J Pain Res. (2025) 18:4109–21. 10.2147/jpr.S535883 40832560 PMC12360397

[11] Sun L Liu K Li M Sun Y Zhu X Chang C. Tracheobronchial amyloidosis accompanied with asthma: a case report and a mini-review. J Asthma Allergy. (2023) 16:1187–93. 10.2147/jaa.S433639 37920270 PMC10619460

[12] Li H Ren X Zhang H Li S He Y Qi T. A clinical study on video bronchoscopy-guided coblation for benign central airway stenosis. Ear Nose Throat J. (2024) 105(10):1455613241235513. 10.1177/01455613241235513 38439622

[13] Virk RS Bansal S Nayak G P L. Plasma ablation-assisted endoscopic management of postintubation laryngotracheal stenosis: an alternate tool for management. Otolaryngol Head Neck Surg. (2019) 161(6):993–5. 10.1177/0194599819881439 31590615

[14] Hao F Yue L Yin X Wang X Shan C. Low-temperature radiofrequency coblation reduces treatment interval and post-operative pain of laryngotracheal recurrent respiratory papillomatosis. Biosci Rep. (2020) 40(5):BSR20192005. 10.1042/bsr20192005 32426815 PMC7251323

[15] Kosnik N Colligan M Neuenschwander M. Tracheobronchial amyloid subglottic stenosis treatment using low-temperature radio frequency therapy. J Bronchology Interv Pulmonol. (2023) 30(3):298–301. 10.1097/lbr.0000000000000893 36004525

[16] Leske V Lazor R Coetmeur D Crestani B Chatté G Cordier JF. Tracheobronchopathia osteochondroplastica: a study of 41 patients. Medicine (Baltimore). (2001) 80(6):378–90. 10.1097/00005792-200111000-00004 11704714

[17] Zhu Y Wu N Huang H-D Dong Y-C Sun Q-Y Zhang W. A clinical study of tracheobronchopathia osteochondroplastica: findings from a large Chinese cohort. PLoS One. (2014) 9(7):e102068. 10.1371/journal.pone.0102068 25013916 PMC4094519

[18] Mangla L Vadala R Kadli SK Prajapat D Talwar D. Tracheobronchial amyloidosis: an uncommon disease with a common presentation. Respirol Case Rep. (2020) 8(7):e00630. 10.1002/rcr2.630 32765884 PMC7396321

[19] Gołębiowski T Kuźniar J Porażko T Wojtala R Konieczny A Krajewska M. Multisystem amyloidosis in a coal miner with silicosis: is exposure to silica dust a cause of amyloid deposition? Int J Environ Res Public Health. (2022) 19(4):2297. 10.3390/ijerph19042297 PMC8871531 35206498

[20] Zhang N Gao Y Zhou Y Gao H Wang H. Bronchoscopic interventional treatment of primary tracheobronchial amyloidosis: analysis of 12 cases. Int J Respir. (2019) 39(14):1086–9. 10.3760/cma.j.issn.1673-436X.2019.14.009 [张楠, 高永平, 周云芝, 高鸿, 王洪武. 支气管镜介入治疗原发性气管支气管淀粉样变性12例分析. 国际呼吸杂志. 2019;39(14):1086-9. doi: 10.3760/cma.j.issn.1673-436X.2019.14.009].

[21] Alloubi I Thumerel M Bégueret H Baste JM Velly JF Jougon J. Outcomes after bronchoscopic procedures for primary tracheobronchial amyloidosis: retrospective study of 6 cases. Pulm Med. (2012) 2012:352719–4. 10.1155/2012/352719 23326661 PMC3544315

[22] Sommer P Kumar G Lipchik RJ Patel JJ. Tracheobronchial amyloidosis managed with multimodality therapies. Ther Adv Respir Dis. (2014) 8(2):48–52. 10.1177/1753465814524470 24594977

[23] Arulanantham J Officer C O'Connor C Baillie T Bass S Williamson JP. Localized tracheobronchial amyloidosis: a rare case presentation and tailored management approaches. Respirol Case Rep. (2021) 9(9):e0820. 10.1002/rcr2.820 34401188 PMC8355433

[24] Sachdeva A Pickering EM Lee HJ. From electrocautery, balloon dilatation, neodymium-doped:yttrium-aluminum-garnet (nd:yAG) laser to argon plasma coagulation and cryotherapy. J Thorac Dis. (2015) 7(Suppl 4):S363–79. 10.3978/j.issn.2072-1439.2015.12.12 26807284 PMC4700359

[25] Reichle G Freitag L Kullmann HJ Prenzel R Macha HN Farin G. Argon plasma coagulation in bronchology: a new method–alternative or complementary? Pneumologie. (2000) 54(11):508–16. 10.1055/s-2000-8254 11132548

[26] Jeong JH Kim J Choi CM Ji W. Clinical outcomes of bronchoscopic cryotherapy for central airway obstruction in adults: an 11-years’ experience of a single center. J Korean Med Sci. (2023) 38(32):e244. 10.3346/jkms.2023.38.e244 37582494 PMC10427217

[27] Chaddha U Agrawal A Kurman J Ortiz-Comino R Dutau H Freitag L. World association for bronchology and interventional pulmonology (WABIP) guidelines on airway stenting for malignant central airway obstruction. Respirology. (2024) 29(7):563–73. 10.1111/resp.14764 38812262

